# Supplementary material for: Dose-response relationships of physical activity with bone mineral density and muscle mass in visceral obesity: A metabolic heterogeneity perspective
Source: PLoS One. 2025 Jul 10;20(7):e0327987. doi: 10.1371/journal.pone.0327987 (PMC12244534; doi:10.1371/journal.pone.0327987)
Supplement: Attachment — Submitted filename: Response to Reviewers.docx [file pone.0327987.s001.docx]

Response Letter to Reviewers’ Comments

Dear reviewers,

Thank you very much for your comments and professional advice. These opinions help to improve the academic rigor of our article. Based on your suggestion and request, we have made the corrected modification to the revised manuscript. In this revised version, changes to our manuscript were all highlighted within the document by using red-colored text. We hope that our work can be improved again. Furthermore, we would like to show the details as follows:

**Responses to comments from reviewer 1:**

**Q1:** The reference number ‘12’ is not complete. Please correct it.

**A:** Thank you for your feedback. This literature has been revised in the article and is recorded as reference 11.

**Q2:** “Loss of bone and muscle mass often indicates accumulation of adipose tissue[9]. Studies in recent years have shown that obese people may co-exist with decreased muscle mass and/or BMD, constituting a complex condition of comorbidities” The relation between obesity and osteoporosis is still debatable. Historically, obesity is protective against osteoporosis. Since this is still an ongoing thesis, further studies are needed, I would revise your statement.

**A:** Thank you for pointing this. There are indeed problems with our original expression. Therefore, we re-describe this part based on the new references 9, 12 and 13. See Lines 78-88 specifically.

**Q3:**  I could not see a clear exclusion criteria. I am wondering if the patients with a history of cancer, or severe comorbidities were included into the study.

**A:** We appreciate the reviewer's concern regarding comorbidities. Our study utilized the NHANES database, which employs a complex sampling design to represent the non-institutionalized U.S. civilian population. To preserve the real-world generalizability of our findings, we did not make further exclusions based on the individual circumstances of the participants.

But as the reviewers suggested, comorbidity is indeed a factor that we have overlooked and may have an impact on the research results. Therefore, we supplemented the data on comorbidity in the study. However, due to the limitations of data entries and missing situations, we made some adjustments based on the Charles Comorbidity Index standard. Specifically as follows:

“The assessment criteria for comorbidities were modified based on the items of the Charles Comorbidities Index[34]. The final items include myocardial infarction, congestive heart failure, stroke, emphysema, chronic pulmonary bronchitis, arthritis, gout, liver disease, diabetes, gastrointestinal diseases, kidney diseases, and cancer or malignant tumors.”

**Q4:**  So, the authors define VAT and SAT and the importance of their ratios. I am wondering in which statistical analysis they used this definition. They used VAI for analysis, but it is totally a different measurement, right?

**A:** Thank you for pointing this. Perhaps it was the ambiguity we expressed that caused the confusion of concepts. In this study, VAT/SAT ratio was used to define visceral obesity per diagnostic criteria. VAI—a composite index incorporating waist circumference, BMI, triglycerides, and HDL—was employed for metabolic clustering to capture lipid-metabolic dysfunction. The former is used to determine the research population, while the latter is used for the analysis of metabolic levels. We chose this method to study whether the metabolic level of people with visceral obesity would mask or even inhibit the beneficial effects of physical activity on bone mineral density and muscle mass.

**Q5:**  Because NHANES is cross-sectional, causality cannot be inferred (e.g., healthier individuals may exercise more). I am wondering what kind of additional data was used for analysis. Any comorbidity scoring index etc.

**A:** We appreciate the reviewer's critical point regarding the inherent limitation of the NHANES cross-sectional design for inferring causality. We fully acknowledge that reverse causality (healthier individuals exercising more) is a valid concern. To mitigate this concern and potential confounding by overall health status, we added the comorbidity statistics modified from the Charles Comorbidity Index to the original model 3. After readjust, the results showed that there was still a significant association between high/moderate levels of physical activity and bone density and muscle mass, which strengthened the argument that physical activity may bring independent benefits.

**Q6:**  Clustering was performed on only 4 variables (SBP, DBP, VAI, HbA1c). I am wondering why only these 4 variables were chosen and whether cluster robustness was tested.

**A:** We appreciate the reviewer's suggestion regarding the potential inclusion of additional markers for a more comprehensive metabolic classification. We acknowledge that factors like detailed lipid profiles and inflammatory markers could theoretically refine the clusters. We considered various metabolic indicators, such as C-reactive protein and postprandial blood glucose, etc. However, in the data structures of different rounds, there are situations where some indicator items are missing, and the proportion of missing values of some indicators is relatively high. Constrained by the structure of the NHANES database and the specific situation of data collection, we ultimately only selected SBP, DBP, VAI, and HbA1c as clustering indicators. Among these four indicators, VAI integrates triglycerides (TG), high-density lipoprotein cholesterol (HDL-C), body mass index (BMI), and waist circumference (WC), making it a comprehensive indicator. In addition, the added blood pressure and blood sugar are also core indicators reflecting the metabolic status. Therefore, we believe that choosing these four indicators is a relatively appropriate decision when the selection of indicators is limited.

Furthermore, regarding the reliability of the clustering results. Through analysis of variance, we found that four metabolic indicators (namely systolic blood pressure, diastolic blood pressure, VAI and glycated hemoglobin) showed highly significant differences among the four metabolic clusters (all analysis of variance P < 0.001) (as shown in Table 4 of our manuscript). It indicates that the clustering process successfully minimizes the differences within groups and maximizes the differences between groups. The study population was successfully divided into different subgroups, which were characterized by different blood pressure levels, visceral fat (captured by VAI), and blood glucose control. The specific characteristics of the clustering groups are presented in Table 4 in the form of mean (standard deviation).

**Q7:**  It is unclear if vitamin D levels, depression scores, and smoking were included in all models. Table 2 mentions these, but the Methods section should explicitly say which covariates are included in which models.

**A:** We appreciate the reviewer's attention to detail regarding covariate specification. We carefully reviewed the content of the original text, the Methods section (subsection 2.6 Statistics Analysis) explicitly states:

“Three sequential models were constructed: Model1 represented the unadjusted crude analysis; Model2 incorporated adjustments for age and sex; and Model3 further adjusted for race, sleep duration, depression, hypertension, TC, VD, smoking and PIR in addition to the covariates in Model2.”

Furthermore, we discovered that due to our negligence, the covariate "education" was not declared here. In fact, this variable has been incorporated into the original research model for adjustment, but it was omitted during the manuscript writing process. We have made adjustments to the relevant content.

**Responses to comments from reviewer 2:**

**Q1:** The detailed and substantial contents of classification about physical activity levels should be described in Methods, although one reference [30] was only cited.

**A:** We sincerely appreciate this suggestion. Perhaps the inaccuracy of the description in the original text has led to the ambiguity of the information on the classification of sports activity levels. Our research approach is to multiply the duration of vigorous recreational activities by 2 and add it to the duration of moderate recreational activities based on the guidelines of the World Health Organization [30] to construct an "equivalent time". Classify the level of physical activity through this "equivalent time".

We have revised the relevant descriptions in the Methods (2.4 physical activity), clarifying the definition of physical activity intensity and the formation method of "equivalent time".

**Q2:** The content of Subgroup analysis seemed ambiguous, which details should be explained clearly.

**A:** Thank you for your feedback. We have revised the **Methods (2.6 Statistics analysis)** to clarify

**Q3:** In this study, the standard methods have not used for the assessment of bone mineral density and muscle mass. This limitation should be described in Discussion.

**A:** Thank you for pointing this. However, we are somewhat confused about the “standard method” mentioned in the suggestion.

If "standard methods" refer to the measurement methods of BMD and muscle mass, the BMD and muscle mass data used in this study were all obtained through Dual-energy x-ray absorptiometry (DXA). This method is currently one of the most widely used imaging techniques, featuring a low radiation dose, high accuracy, and ease of use. And compared with CT and MRI, DXA has very good accuracy in fat treatment and lean mass.

If the "standard method" refers to the method for evaluating BMD and muscle mass quality levels, the ALM/BMI method mentioned by the Foundation for the National Institutes of Health was adopted in this study to evaluate muscle mass. The reason for choosing this indicator is just as described in Literature 27. ALM adjusted for BMI can more accurately reflect the relative insufficiency of muscle mass relative to body size. It can solve the problem of masking insufficient muscle mass when an individual is obese. The T-score is used to evaluate BMD, and the reference standards levels for evaluating BMD T-score also come from Americans.

However, in the population with visceral obesity, the muscle mass adjusted by BMI may not be able to distinguish the distribution of fat in the body. This limitation does exist, and we have supplemented this in the discussion. See Lines 482-484 specifically.

We welcome and appreciate any further suggestion to help us improve our understanding and methodology.

**Q4:** The results of this study should be discussed in association with visceral obesity.

**A:** Thank you for your feedback. We reorganized the sequence and logic of the Discussion part and discussed the research results from the perspective of visceral obesity.

**Responses to comments from reviewer 3:**

**Q1:** PA levels were categorized as inactive (0 min/week), low-active (1-150), moderate-active (150-300), and high-active (>300) in this study. When examining the relationship between PA and BMD/muscle mass, it is important to consider the type of exercise such as aerobic exercise, resistance exercise, or combined exercise, as well as its intensity and duration. The details are unclear in surveys of PA based on self-reported questionnaires of this cross-sectional study. The issue with this paper is that, given the wide variety of available exercise options, it lacks specific recommendations that can be suggested to the patients.

**A:** Thank you for your feedback. We appreciate the reviewer's insightful comment regarding the importance of exercise modality specificity. We acknowledge that differentiating between aerobic, resistance, and combined exercise would enhance clinical applicability. However, the NHANES physical activity questionnaire (GPAQ) captures only total moderate-to-vigorous recreational physical activity (MVPA) duration for adults. Crucially, **NHANES does not collect modality-specific activity data (e.g., resistance vs. aerobic) for its adult cohort** — such granularity is exclusively available in youth-specific survey modules. Given this inherent limitation in the publicly available dataset, our analysis focused on the dose-response relationship of total MVPA with musculoskeletal outcomes.

**Q2:** Subgroup analyses were conducted based on metabolic clusters, which were defined using k-means clustering according to blood pressure, visceral adiposity index, and HbA1c levels. Although metabolic clusters are being classified using blood pressure, visceral fat index, and HbA1c levels, is this classification valid? How do the authors think about the opinion that a classification based on more comprehensive data including lipid profiles and inflammatory markers yield more accurate and valid results?

**A:** We appreciate the reviewer's insightful suggestion that incorporating additional metabolic markers could enhance cluster precision. We fully acknowledge that such expansion might provide a more granular characterization of metabolic heterogeneity.

Regarding the reliability of the clustering results, through analysis of variance, we found that there were extremely significant differences among the four metabolic indicators (namely systolic blood pressure, diastolic blood pressure, VAI and glycated hemoglobin) among the four metabolic clusters (all analysis of variance P < 0.001, see Table 4 of the paper for details). This indicates that the clustering process successfully achieved the minimization of intra-group differences and the maximization of inter-group differences. The study population was successfully divided into different subgroups, which were characterized in terms of blood pressure levels, visceral fat (reflected by VAI), and blood glucose control. The specific characteristics of the clustering groups are presented in Table 4 in the form of mean (standard deviation).

During the research process, we also considered a variety of metabolic indicators, including C-reactive protein, postprandial blood glucose, etc. However, due to the differences in data structures among different rounds, some indicator items are missing, and the proportion of missing values for some indicators is relatively high. Limited by the structure of NHANES database and the specific circumstances of data collection, only systolic blood pressure (SBP), diastolic blood pressure (DBP), visceral fat index (VAI), and glycated hemoglobin (HbA1c) were ultimately selected as clustering indicators. Among them, VAI integrates information such as triglycerides (TG), high-density lipoprotein cholesterol (HDL-C), body mass index (BMI), and waist circumference (WC), and is a comprehensive indicator. In addition, blood pressure and blood sugar are also core indicators reflecting metabolic conditions. Therefore, in the case where the selection of indicators is limited, we believe that choosing these four indicators is a relatively appropriate decision.

**Q3:** Is whether or not patients are receiving treatment for hypertension, dyslipidemia, or diabetes mellitus taken into consideration in this analysis?

**A:** Thank you for your feedback. We analyzed the medication situation and found that the proportion of missing values was relatively high (including 2321 missing values for hypertension medication and 2572 missing values for hyperlipidemia medication) in this study. Therefore, we did not include whether medication was taken as an adjusting variable in the study.

According to the editor and reviewers’ comments, we have made extensive modifications to our manuscript to make our results convincing. Thank you again for your positive comments and valuable suggestions to improve the quality of our manuscript.
